# Supplementary material for: Frequency, clinical characteristics and outcomes of Tropidolaemus species bite envenomations in Malaysia
Source: PLoS Negl Trop Dis. 2023 Jan 4;17(1):e0010983. doi: 10.1371/journal.pntd.0010983 (PMC9812298; doi:10.1371/journal.pntd.0010983)
Supplement: S1 Table — (PDF) [file pntd.0010983.s001.pdf]

SUPPORTING INFORMATION

Supplementary Material S1

S1 Table. The number of *Tropidolaemus* spp. bite cases consulted to RECS from each district and state in Malaysia from 2015-2021.

State	District	Number of Incident	Total
**Johor**	Batu Pahat	0	1
	Johor Bahru	0	
	Kluang	0	
	Kulai Jaya	0	
	Ledang	0	
	Mersing	0	
	Muar	0	
	Pontian	0	
	Segamat	0	
	Kota Tinggi	1	
**Kedah**	Alor Setar	0	0
	Baling	0	
	Bandar Baharu	0	
	Kota Setar	0	

	Kuala Muda	0	
	Kuala Pasu	0	
	Kubang Pasu	0	
	Kulim	0	
	Langkawi	0	
	Padang Terap	0	
	Pendang	0	
	Sik	0	
	Yan	0	
**Kelantan**	Bachok	0	2
	Gua Musang	0	
	Jeli	0	
	Kota Bharu	0	
	Kuala Krai	0	
	Machang	0	
	Pasir Puteh	0	
	Tanah Merah	0	
	Tumpat	0	
	Pasir Mas	2	
**Kuala Lumpur**	Kuala Lumpur	0	0

**Melaka**	Alor Gajah	0	1
	Masjid Tanah	0	
	Melaka Tengah	0	
	Jasin	1	
**Negeri Sembilan**	Jelebu	0	7
	Rembau	0	
	Seremban	0	
	Tampin	0	
	Jempol	1	
	Kuala Pilah	1	
	Port Dickson	5	
**Pahang**	Bera	0	17
	Cameron Highlands	0	
	Maran	0	
	Pekan	0	
	Temerloh	0	
	Timur Laut	0	
	Jerantut	1	
	Rompin	1	
	Bentong	2	
	Kuantan	2	
	Raub	3	
	Lipis	8	

**Perak**	Hilir Perak	0	63
	Kerian	0	
	Kuala Perak	0	
	Manjung	0	
	Pangkor	0	
	Perak Tengah	0	
	Hulu Perak	6	
	Kinta	8	
	Batang Padang	9	
	Kampar	11	
	Kuala Kangsar	11	
	Larut Matang	18	
**Perlis**	Abi	0	0
	Arau	0	
	Beseri	0	
	Bintong	0	
	Chuping	0	
	Kangar	0	
	Kecur	0	
	Kuala Perlis	0	
	Kurong Batang	0	
	Ngolang	0	
	Paya	0	

	Sanglang	0	
	Seriab	0	
	Sungai Adam	0	
	Titi Tinggi	0	
	Utan Aji	0	
**Selangor**	Kuala Langat	0	13
	Petaling	0	
	Sabak Bernam	0	
	Sepang	0	
	Hulu Langat	1	
	Klang	1	
	Kuala Selangor	1	
	Gombak	5	
	Hulu Selangor	5	
**Terengganu**	Hulu Terengganu	0	9
	Kuala Nerus	0	
	Kuala Terengganu	0	
	Marang	0	
	Setiu	0	
	Besut	2	
	Dungun	2	
	Kemaman	5	

**Pulau Pinang**	Seberang Perai Utara	0	7
	Timur Laut	0	
	Seberang Perai Selatan	2	
	Seberang Perai Tengah	2	
	Barat Daya	3	
**Sabah**	Penampang	0	40
	Pensiangan	0	
	Pitas	0	
	Semporna	0	
	Tambunan	0	
	Telupid	0	
	Tenom	0	
	Tongod	0	
	Kinabatangan	0	
	Kunak	0	
	Labuan	0	
	Labuk Sugut	0	
	Lahad Datu	0	
	Nabawan	0	
	Beaufort	1	
	Beluran	1	
	Keningau	1	
	Kota Marudu	1	

	Papar	1	
	Tawau	2	
	Kota Belud	3	
	Kudat	3	
	Ranau	3	
	Sandakan	3	
	Sipitang	3	
	Tuaran	4	
	Kuala Penyu	6	
	Kota Kinabalu	8	
**Sarawak**	Belaga	0	150
	Daro	0	
	Julau	0	
	Lubok Antu	0	
	Matu	0	
	Meradong	0	
	Pakan	0	
	Samarahan	0	
	Saratok	0	
	Selangau	0	
	Song	0	
	Tatau	0	
	Asajaya	0	
	Dalat	1	

Kanowit	1
Mukah	1
Lundu	2
Sibu	2
Simunjan	2
Betong	3
Kapit	4
Sarikei	5
Lawas	9
Limbang	8
Marudi	8
Bau	11
Sri Aman	11
Bintulu	15
Kuching	18
Miri	21
Serian	28
