# Supplementary material for: Frequency, clinical characteristics and outcomes of Tropidolaemus species bite envenomations in Malaysia
Source: PLoS Negl Trop Dis. 2023 Jan 4;17(1):e0010983. doi: 10.1371/journal.pntd.0010983 (PMC9812298; doi:10.1371/journal.pntd.0010983)
Supplement: Attachment — Submitted filename: responses to reviewers.docx [file pntd.0010983.s002.docx]

Dear editors,

Thank you for the opportunity to response to the reviewers’ questions. Please find below the detailed list of each of the reviewers’ questions. The description of changed made has also been tracked change in the manuscript.

**Responses to reviewers’ Questions**

**Methods**

Reviewer #1: The manuscript describes the incidence of envenomation by Tropidolaemus snakes in Malaysia, during the period of 2015 to 2021. Signs and symptoms, as well as the outcomes of these envenomation are also described. The authors clearly pointed out the aim of the study and describe the affected population in appropriated tables and figures.

**Author’s response :** Thank you for the kind observation.

Reviewer #2: -Are the objectives of the study clearly articulated with a clear testable hypothesis stated?
Yes
-Is the study design appropriate to address the stated objectives?
yes
-Is the population clearly described and appropriate for the hypothesis being tested?
yes
-Is the sample size sufficient to ensure adequate power to address the hypothesis being tested?
Yes
-Were correct statistical analysis used to support conclusions?
NA
-Are there concerns about ethical or regulatory requirements being met?

They need to clarify how did they identify the species of snakes.

**Author’s response :** Thank you for the kind observation. The diagnosis of each case was based on the actual snake brought to the hospital for identification or the pictures of the actual snake that caused the bite incidence. The diagnosis of each case consulted to RECS were verified by the experts in the field including the verification of the snake species. This has been included in the methods line 117 – 118.

Reviewer #3: The objectives of the study were not clearly articulated with the hypothesis stated, as identification of the Tropidolaemus species was not clearly detailed. This aspect is a key point and should be completed. As there are more than 200 species of snakes in Malaysia, is there any possibility of no venomous snakes to mimic the behavior or the appearance of Tropidolaemus species snakes, including the ontogenetic variability in the morphology? This would be important to understand in which extend such variations could contribute to a misdiagnosed snakebite envenoming or not.
Another limitation of the study is related to the dataset, composed of cases reported remotely. In this situation, it should be clearly stated how authors ensured precise identification of the causative agent of the injuries.

**Author’s response :** Thank you for the kind observation. The diagnosis of each case was based on the actual snake brought to the hospital for identification or the pictures of the actual snake that cause the bite incidence. The diagnosis of each case consulted to RECS were verified by the experts in the field including the verification of the snake species. This has been included in the methods line 117 – 118.

**Results**

Reviewer #1: The manuscript presents the results in an appropriate number of tables and figures.

**Author’s response :** Thank you.

Reviewer #2:

-Does the analysis presented match the analysis plan?
-Are the results clearly and completely presented?
Yes, but they need to better discuss their findings.
-Are the figures (Tables, Images) of sufficient quality for clarity?
yes, but can be improved.

**Author’s response :** Thank you.

Reviewer #3: In general, snakebite envenoming is considered an occupational event during agricultural activities. In the present study, however, most cases were referred as non-occupational injuries. Could authors describe in which circumstances the cases occurred? Were these conditions compatible with Tropideolaemus behavior characteristics?

**Author’s response :** The circumstances of bite incidence were only classified into ‘occupational related’ and ‘non-occupational related’ based on the documentation of each case during RECS consultation. The compatibility of the behavior of Tropidolaemus species has been discussed in the discussion segment ( line 182 – 198 )

A full map of the country would be more illustrative of the geographical distibution of the two Tropidolaemus species. A more exhaustive discuss of the dicotomic distribution of T. wagleri and T. subannulatus in the two Malaysian states is missing.

**Author’s response :** Thank you for the input, however, the geographical distribution map in Malaysia is clearly demonstrating the locality of *Tropidolaemus wagleri* in Peninsular Malaysia and *Tropidolaemus subannulatus* is in East Malaysia. This has been explicitly stated in line 64 – 68. The reason why the two states (Kedah and Perlis) in Peninsular Malaysia appears to be missing from the geographical distribution is because this study only analyse the data from RECS consultation. The state of Kedah and Perlis already have the connection to RECS, however no cases of *Tropidolaemus wagleri* bites were consulted to RECS during the study period. This does not mean there were no *Tropidolaemus wagleri* present in both states. The consultation to RECS is not obligatory and is up to the physicians’ prerogative to consult RECS or not for each case.

As there is no specific antivenom for Tropidolaemus envenoming available, which antivenom(s) is/are available in Malaysia for snakebite envenoming? Give the name of the manufacturer(s), characteristics of the product (especially neutralizing potency), doses indication, etc. Is there any study to assess the cross neutralization between antivenom(s) and T. subannulatus or T. wagleri snake venoms to justify the use in the country?

**Author’s response :** There is no antivenom developed for Tropidolaemus species as it did not meet the WHO’s criteria for antivenom production. Malaysia do not manufacture antivenom, however due to the closely related medically significant venomous land snake species to Thailand, the country resort to import the appropriate antivenom from Queen Saovabha Memorial Institute (Thai Red Cross). This is explained in Introduction line 95 – 99, together with the appropriate references.

Although sometimes expressed as the number of cases during a given period of time, by definition, incidence is a measure of the frequency or probability of occurrence of a given medical condition in a population within a specified period of time. No incidence rates were herein calculated, thus the term should be avoided, either in the text or table. Instead of incidence, prefer frequency.

**Author’s response :** Thank you. This has been amended accordingly.

Outcomes were not clearly described for those who stayed at the hospital more than 24hrs (61 cases), as it does not correspond to those with severe (14) and moderate (69) pain. Were there refractory cases to analgesics?

**Author’s response :** No, there is no documentation about any refractory cases to analgesics. All cases responded to the analgesia given and resolved over time and with some did not require hospitalization more than 24 hours.

**Conclusion**

Reviewer #1: The conclusion is generic and not directly related to the results presented.

**Author’s response :** Thank you for the comment. The conclusion has been improved accordingly.

Reviewer #2: -Are the conclusions supported by the data presented?
Yes,

-Are the limitations of analysis clearly described?
Limitations are not fully in line with the aim of this work
-Do the authors discuss how these data can be helpful to advance our understanding of the topic under study?
Not quite.
-Is public health relevance addressed
Somehow, authors should give more emphasis bout this.

**Author’s response :** Thank you for the comment. The conclusion has been improved accordingly. The public health relevance has been added in the Discussion line 212 – 223.

Reviewer #3: Conclusions are not strongly supported by the data presented as case definition of Tropidolaemus snakebite envenoming is not clearly stated. The remote identification of the two species constitutes a major limitation of the analysis. Authors suggest a revision in the WHO Category listing, which is premature, considering the limitation in the methodology of the study.

**Author’s response :** Thank you for the comment. This is exactly why this study is important because there is no clinical evidence to suggest why *Tropidolaemus subannulatus* is currently included in the WHO Category 2 list of medically important venomous snake. It is definitely premature for WHO to include *Tropidolaemus subannulatus* in the Category 2.


**Editorial and Data Presentation Modifications**

Reviewer #1: (No Response)

Reviewer #2: Some paragraphs should be rephrased in order to improve readability and clarity.

**Author’s response :** Thank you.

Reviewer #3: (No Response)

--------------------

**Summary and General Comments**

Reviewer #1: The manuscript is of importance, despite describing accidents caused by a snake with a low incidence of envenoming between snakebites in Malaysia, and with less severe intensity.

**Author’s response :** Thank you for the comment. To clarify, it is not a low incidence of envenoming, rather, it is not a significant injury caused by venom from *Tropidolaemus* species in human. A different manifestation of envenomation may occur in other prey animals such as rodents or birds.

Reviewer #2:

The manuscript ” Incidence, clinical characteristics and outcomes of Tropidolaemus species bite envenomation in Malaysia” (PNTD-D-22-01125) compiles the number of Tropidolaemus bites that were referred to the Remote Envenomation Consultation Services (RECS) for a period of six years. According to them, although T. subannulatus and T. wagleri are associated with high morbidity in the area, the use of antivenom to treat these snakebites is not prescribed. In addition, authors provide additional information ranging from the morphological characteristics of these snakes, seasons with the highest morbidity, demographics of patients, site of the bites and the typical first aid performed, e.g.

**Author’s response :** Thank you for the comment. However, the authors did not report high morbidity associated with *Tropidolaemus wagleri* or *Tropidolaemus subannulatus* in Malaysia. Almost all of the cases were only presented with mild local symptoms with no recorded permanent morbidity or mortality.

This manuscript can be enhanced if authors review, analyze and discuss additional data regarding the venom from Tropidolaemus , its biochemistry, pharmacology and the way is neutralized. Is there any specific or para-specific antivenom? If there were a person with a high degree of envenomation what antivenom should be administer?

**Author’s response :** Thank you for the comment. It is not the objective of this study to analyse biochemistry and pharmacology of Tropidolaemus venom and neutralization by existing antivenom. This has been referred to in the Introduction line 88 – 99.

**Particular comments,**

Author Summary

Line 45-47.- What is the purpose of excluding T. subannulatus from WHO category 2? Is this highly venomous or associated to high mortality? Please explain

**Author’s response :** There is no clinical evidence for including *Tropidolaemus subannulatus* in the WHO Category 2. This study provides the necessary clinical evidence that WHO Category 2 is looking for. According to WHO Guidelines for the Production, Control and Regulation of Snake Antivenom Immunoglobulins, the medically important venomous snakes are divided into two categories: Category 1 and Category 2. Snakes that fall into Category 1 are venomous snakes which are common or widespread and causes numerous snakebites that contributes to high levels of morbidity, disability and mortality. Meanwhile, Category 2 is venomous snakes that are capable of causing morbidity, disability and mortality, in which the exact clinical data are still lacking and less frequently implicated. The snakes that are listed in both categories are species for which antivenom is indicated. In the WHO category listings, *T. subannulatus* is one of the snakes in Malaysia that falls into Category 2, which indicates *T. subannulatus* being in high priority for indication of antivenom.

Introduction.
Line 52.- How mechanical injury can be fatal? Please, explain or amend.

**Author’s response. :** Mechanical injury can be fatal from a bite, example from a Python either directly or indirectly.

Line 52-55- How envenomation happens when venom is sprayed into the eyes? Please, explain or amend.

**Author’s response :** Thank you for the comment. We have amended in line 51 – 53.

Line 59-60 .- Lack of cohesivity between these two paragraphs.

**Author’s response :** The two paragraphs are separated.

Line 60-62.- Authors are saying that there is a limited epidemiological data, but they provide exact numbers in previous and further paragraphs. Please, clarify.

**Author’s response :** The limited epidemiological data is pertaining to Malaysia. The numbers quoted were disjointed from various sources. That is what meant by the word ‘limited’.

In the text:” During the same period, there were 16 deaths averaging 3 to 4 fatalities per year” what species and what is the source of information? Please cite.

**Author’s response** : These figures were cited from reference no.2, Ministry of Health Malaysia P. Guideline: Management of Snake bite. Malaysia: Ministry of Health Malaysia, Putrajaya 2017. No causative snakes species were mentioned, thus emphasizing the poor and limited epidemiological data mentioned above.

First and second paragraph could be merged to make one cohesive and concise text.

**Author’s response** : Thank you for the comment. We have merged the two paragraphs.

Line 73-75. “There are two subspecies of Tropidolaemus in Malaysia, T. wagleri and T. subannulatus. T. wagleri is indigenous to Peninsular Malaysia, WHEREAS T. subannulatus is indigenous to Borneo”. In here you have a distribution map, this could also be referred as Fig 1 or 2.

**Author’s response** : Thank you for the suggestion. However, it is inappropriate to refer result’s figures in Introduction. The references for such have been appropriately cited.

Line 93-103. Behavior and distribution of this snake are important. However, authors should focused more on providing as much information as available about venom composition, its biochemistry and antivenoms, rather than describing physical characteristics of these species.

**Author’s response** : Thank you for the suggestion. It is not the objective of this study to analyse biochemistry and pharmacology of *Tropidolaemus* venom and neutralization by existing antivenom. This has been referred to in the Introduction line 89 – 100. The references for such have been appropriately cited.

Line 106-118. In the last paragraph authors provide information that does not match with the aim of the work. Please re-write and make it sound with the objectives, findings and conclusions of it.

**Author’s response :** Thank you for the comment. The appropriate paragraphs have been inserted.

Methods.
Line 106-118. What is the piece of evidence confirming that the envenomation was done by Tropidolaemus species and not by any other ? In this regard, how patients or physicians identified at the genus and species level? Please clarify and state that in the corresponding section.

**Author’s response :** Thank you for the observation. The diagnosis of each case was based on the actual snake brought to the hospital for identification or the pictures of the actual snake that caused the bite incidence. The diagnosis of each case consulted to RECS were verified by the experts in the field including the verification of the snake species. This has been included in the methods line 117 – 118.


Results
 Line 136- 140. Again, how did they confirm the species?

**Author’s response :** Thank you for the observation. The diagnosis of each case was based on the actual snake brought to the hospital for identification or the pictures of the actual snake that caused the bite incidence. The diagnosis of each case consulted to RECS were verified by the experts in the field including the verification of the snake species. This has been included in the methods line 117 – 118.


Line 159. Figure 3. Not many people know Malaysian geography, it would be great to indicate as A and B the name of these two regions and write down the name (in the figure or figure description) of the districts where you found the highest incidence of these two species.

**Author’s response :** The appropriate changes has been made in Figure 3 and the legend for Figure 3.

Line 172-173.- site of the bite instead of “puncture wound”
**Author’s response :** The appropriate changes has been made in the paragraph line 168 and in Table 2.

Line 178.- Table 2 description is not clear. Please re-phrase it.

**Author’s response :** The appropriate changes has been made in Table 2 and Table 2 description.

Line 185.- what is Cloxacillin? Indicate. If this was undocumented how did you get this information?
**Author’s response** : Cloxacillin is an antibiotic. It was not documented in the RECS consultation data for the two cases.

Line 187-188.- Again, how undocumented information became available for authors?

**Author’s response** : This data was obtained from RECS consultation log in which the consultation was initially established through telephone calls, email and various forms of short text messaging systems. Smart mobile communication device is now essential for the consultation procedure. Various consultation groups have been created on *WhatsApp* exclusively for Doctors and Pharmacists from various healthcare facilities. All consultation procedures strictly follows the guideline provided by the MOH Director General of Health circular bil. 10/2016. The inclusion of AI technology into the recent development of MyRECS Mobile App will revolutionise RECS consultation protocol in the near future.

 “T. wagleri gets its common name, Temple pit viper from The Snake Temple in Penang, Malaysia. This was built to honour Chor Soo Kong, a Buddhist monk from the Song Dynasty, for his deeds in healing the sick and giving shelter to snakes. After the temple was built, snakes started taking shelter in the temple, particularly T. wagleri ” This information is interesting. Nonetheless, authors should focus on more relevant data like venom composition, antivenoms, WHO information, public health management or even awareness or educational programs.

**Author’s response** : Thank you for the comment. It is not the objective of this study to analyse biochemistry and pharmacology of Tropidolaemus venom and neutralization by existing antivenom. This has been referred to in the Introduction line 88 – 99. The public health relevance has been added in the Discussion line 212 – 223.

Line 224-225. This is a good opportunity to talk about venom composition.

**Author’s response** : Thank you for the comment. It is not the objective of this study to analyse biochemistry and pharmacology of Tropidolaemus venom and neutralization by existing antivenom. This has been referred to in the Introduction line 88 – 99.

Line 232-233 This is a good opportunity to talk about specific antivenoms, availability, specificity, Etc.

**Author’s response** : Thank you for the comment. It is not the objective of this study to analyse biochemistry and pharmacology of Tropidolaemus venom and neutralization by existing antivenom. This has been referred to in the Introduction line 88 – 99.

Conclusion

-Authors mention “ Public awareness regarding snake bite is important to reduce the incidence of snake bite and its outcomes” . Please discussed why in the corresponding section.

**Author’s response** : because as explained in the Discussion segment, inappropriate first aid intervention was still widely practice in the community. Therefore, public awareness will inculcate the culture of safety-seeking behavior (prevention is better than cure). This will indirectly also reduce the frequency of snake bite in Malaysia in line with the WHO roadmap (strategy) to prevent and control snakebite envenoming with the aim to halve the numbers of deaths and cases of disability due to snakebite envenoming by 2030 through a programme that targets affected communities and their health systems, and by ensuring access to safe, effective treatment through increased cooperation, collaboration and partnership at all levels (WHO).

-“We suggest that human envenomation from Tropidolaemus spp. does not require antivenom under normal circumstances” what is a normal circumstance? What kind of antivenom is recommended?

**Author’s response** : There is no antivenom recommended for *Tropidolaemus spp.* bite envenoming. We have amended accordingly in the Conclusion.

- “Symptomatic clinical management with analgesia and short period of close serial monitoring are sufficient to manage these patients.” Why do you recommend this? Based on what study? Did authors measure the lethality of this venoms? Does it contain non-dangerous toxins?

**Author’s response** : This recommendation is based on best clinical practice for managing snake-related injury for Malaysia. The findings of this study further support such clinical practices for *Tropidolaemus spp.* bite. There is no study on the lethality of the venom from *Tropidolaemus spp.* in human. Our study outcome did not record any fatality incidence from *Tropidolaemus spp.* bite. The venom may contain dangerous toxins for murine and avian as described from previous toxinology studies which were referred to in this study.

References,

Please, Italicized scientific names and cite according to journals criteria.

**Author’s response** : Changes have been made accordingly. References is according to Vancouver style.

Reviewer #3: (No Response)

--------------------

PLOS authors have the option to publish the peer review history of their article (<a href="<https://journals.plos.org/plosntds/s/editorial-and-peer-review-process#loc-peer-review-history>" target="_blank">what does this mean?</a>). If published, this will include your full peer review and any attached files.<br><br>

If you choose “no”, your identity will remain anonymous but your review may still be made public.<br><br>

<b>Do you want your identity to be public for this peer review?</b> For information about this choice, including consent withdrawal, please see our <a href="<https://www.plos.org/privacy-policy>" target="_blank">Privacy Policy</a>.

Reviewer #1: No

Reviewer #2: No

Reviewer #3: No


Figure Files:
While revising your submission, please upload your figure files to the Preflight Analysis and Conversion Engine (PACE) digital diagnostic tool, [https://pacev2.apexcovantage.com](https://pacev2.apexcovantage.com/). PACE helps ensure that figures meet PLOS requirements. To use PACE, you must first register as a user. Then, login and navigate to the UPLOAD tab, where you will find detailed instructions on how to use the tool. If you encounter any issues or have any questions when using PACE, please email us at [figures@plos.org](mailto:figures@plos.org).

Data Requirements:
Please note that, as a condition of publication, PLOS' data policy requires that you make available all data used to draw the conclusions outlined in your manuscript. Data must be deposited in an appropriate repository, included within the body of the manuscript, or uploaded as supporting information. This includes all numerical values that were used to generate graphs, histograms etc.. For an example see here: <http://www.plosbiology.org/article/info%3Adoi%2F10.1371%2Fjournal.pbio.1001908#s5>.

Reproducibility:
To enhance the reproducibility of your results, we recommend that you deposit your laboratory protocols in [protocols.io](http://protocols.io/), where a protocol can be assigned its own identifier (DOI) such that it can be cited independently in the future. Additionally, PLOS ONE offers an option to publish peer-reviewed clinical study protocols. Read more information on sharing protocols at <https://plos.org/protocols?utm_medium=editorial-email&utm_source=authorletters&utm_campaign=protocols>

__________________________________________________
In compliance with data protection regulations, you may request that we remove your personal registration details at any time. (Use the following URL: <https://www.editorialmanager.com/pntd/login.asp?a=r>). Please contact the publication office if you have any questions.
